# Prevalence, Antibiotic Resistance and Virulence of *Enterococcus* spp. Isolated from Traditional Cheese Types

**DOI:** 10.4314/ejhs.v32i4.17

**Published:** 2022-07

**Authors:** Ali Jahansepas, Mohammad Aghazadeh, Mohammad Ahangarzadeh Rezaee, Siamak Heidarzadeh, Jalal Mardaneh, Alireza Mohammadzadeh, Omid Pouresmaeil

**Affiliations:** 1 Reference Laboratory, Imam Khomeini Hospital, Urmia University of Medical Sciences, Urmia, Iran; 2 Infectious and Tropical Diseases Research Centre, Tabriz University of Medical Sciences, Tabriz, Iran; 3 Department of Clinical Microbiology, Faculty of Medicine, Tabriz University of Medical Sciences, Tabriz, Iran; 4 Department of Microbiology and Virology, School of Medicine, Zanjan University of Medical Sciences, Zanjan, Iran; 5 Department of Microbiology, School of Medicine, Infectious Diseases Research Center, Gonabad University of Medical Sciences, Gonabad, Iran; 6 Department of Microbiology and Virology, Faculty of Medicine, Mashhad University of Medical Sciences, Mashhad, Iran; 7 Student Research Committee, Faculty of Medicine, Mashhad University of Medical Sciences, Mashhad, Iran

**Keywords:** Traditional cheese, Virulence factors, E. faecium, E. faecalis, Antibiotic resistance

## Abstract

**Background:**

Enterococci are naturally found in the gastrointestinal (GI) tract of animals and humans, as well as animal-derived foods and vegetables. We here aimed to determine the prevalence, antibiotic resistance, and virulence determinants of E. faecium and E. faecalis in traditional cheese in the North-west of Iran.

**Materials and Methods:**

Fifty specimens of popular traditional cheese from dairy stores of Urmia and Tabriz, Iran, were collected. Identification of the genus and species of enterococci was done using molecular and phenotypic techniques.

**Results:**

Forty-eight (96 %) of 50 traditional cheese samples were harboring Enterococcus spp, including Enterococcus faecalis (n= 40; 83.33 %) and E. faecium (n= 8; 16.67 %). The prevalence of enterococci ranged from 1.1×10^5^ to 9.7×10^4^ CFU/g, and 1.1×10^3^ to 9.8×10^3^ CFU/g in Urmia and Tabriz samples, respectively. Rifampicin resistance (n= 38; 79.2 %) was the most common pattern observed in the susceptibility test, which was followed by quinupristin/dalfopristin (n= 33; 68.75 %). Among E. faecalis isolates, cpd (100 %), ace (92.5 %) and gelE (87.5 %), and among E. faecium isolates, gelE (100 %) and asa1 (75 %) were found to have the most common virulence genes.

**Conclusion:**

E. faecalis was the predominant species, displaying more virulence determinants. It also had high antibiotic resistance, as compared to E. faecium. The enterococci identified here commonly expressed virulence and antibiotic resistance determinants. So, it is required to improve the maintenance and production quality of traditional cheese to avoid enterococci contamination.

## Introduction

Animals and humans' gastrointestinal (GI) tract commonly harbor a variety of bacteria, including enterococci. These bacteria are also present in various animal-derived foods such as cheese, milk, and meat, as well as in vegetables. This widespread distribution of enterococci can be attributed to their capability to adapt to and endure different inhabitants (1). These bacteria are widespread in dairy products such as raw milk and traditional types of cheeses ( the product of fermentation of unpasteurized milk ) (2, 3). The increasing global population, which is turning to a global health issue, requires a leap in the production of dairy products. On the other hand, consuming raw milk cheese can be a route of transferring of various microorganisms to the human body such as bacteria, including lactic acid bacteria, *Leuconostoc* Spp., Streptococci, staphylococci, corynebacterial, *Micrococcus* Spp., *E. coli* and *Enterococcus* Spp. (3, 4). The raw dairy product seems to be contaminated with enterococcal strains from various sources (e.g., environment, animals, humans) during the production process (5).

*E. faecium* and *E. faecalis* have been reported as the most common *Enterococcus* spp. identified in cheese; nevertheless, a variety of other enterococci strains may be seen in this product (6). Some enterococci strains are even used as starter cultures in cheese production industries, which is a source of the enterococci isolated from cheese (2). Nonetheless, enterococci bacteria are also known as emerging pathogenic agents causing human diseases. Examples of serious infections like urinary tract infections (UTIs), bacteraemia, and endocarditis have been shown to be associated with enterococci species (7).

A main pathogenic feature of enterococci is their emerging resistance to various antibiotics (8), contributing to the rising incidence of enterococci-associated nosocomial infections. Multiple virulence determinants such as plasmid-encoded pheromones, producing biofilms, expressing various host adhesion molecules and proteins, aggregation molecules, cytolysins, and some enzymes such as hyaluronidase, serine proteases, and gelatinase have been reported in enterococci species (9,10). Although some studies have already been conducted on the microbiota diversity of typical Iranian dairy products, such as Lighvan, Koozeh, and Tarkhineh (11,12), no information is yet available regarding the prevalence, antibiotic resistance and virulence of enterococci present in traditional cheese types in the North-west of Iran. Therefore, this study was aimed to determine the prevalence, the antibiotic susceptibility patterns and virulence determinants of *Enterococcus* spp., especially *E. faecalis* and *E. faecium* obtained from traditional cheese types in the North-west of Iran.

## Materials and Methods

**Sampling**: Cheese samples (n=50) produced from cow or ewe milk through traditional methods were purchased from random dairy product stores in Tabriz (25 samples) and Urmia (25 samples), northwestern Iran. The presence of enterococci bacteria in these samples was investigated after transferring them to a laboratory in ice bags.

**Isolation and enumeration of *Enterococcus* spp.**: *Enterococcus* spp. were isolated and counted as previously described (13). Initially, peptone water (0.1 % (wt/vol), 225 mL) (Merck, Co., Germany) was used to homogenize 25-gram pieces of the samples. After preparing 10-fold serial dilutions of 0.1 % (wt/vol) peptone water and the homogenates, a specific volume of each dilution (100 µL) was placed on the surface of the plates containing bile esculin (BA) agar and kanamycin aesculin azide (KAA) agar (Merck, Co., Germany). After incubation at 37 °C for 24 h, *Enterococcus* spp. were presumptively enumerated on the plates that had at least 25 to 250 colonies. According to the square root of the total number of colony forming units (CFU), a certain number of colonies per each plate were subjected to genotypic and phenotypic analyses.

***Enterococcus* presumptive characterization**: Enterococci presumptive characterization at both species and genus levels (*E. faecium* and *E. faecalis*) was done based on the standard biochemical tests, as previously described (14, 15).

**Multiplex PCR identification**: The genera of enterococci were identified based on the amplification of *rrs* (16SrRNA) gene using specific primers. Specific primers were also used to characterize *ddl* gene to confirm the identification of *E. faecium* and *E. faecalis* (Table 1). Total DNA extraction and multiplex PCR were assayed as previously described (16,17).

**Table 1:** Primers used in this study

Genes	Sequence (5′ to 3′)	Annealing	Amplicon Size (bp)	Ref
***rrs* (16SrRNA)**	F1: GGATTAGATACCCTGGTAGTCC	54	320	(16)
	R1: TCGTTGCGGGACTTAACCCAAC			
***ddl_E. faecalis_***	F2: ATCAAGTACAGTTAGTCTTTATTAG	54	941	(17)
	R2: ACGATTCAAAGCTAACTGAATCAGT			
***ddl_E. faecium_***	F3: TTGAGGCAGACCAGATTGACG	54	658	(17)
	R3: TATGACAGCGACTCCGATTCC			
***asa*1**	F4: GCACGCTATTACGAACTATGA	56	375	(17)
	R4: TAAGAAAGAACATCACCACGA			
***gel*E**	F5: TATGACAATGCTTTTTGGGAT	56	213	(17)
	R5: AGATGCACCCGAAATAATATA			
***esp***	F6: AGATTTCATCTTTGATTCTTGG	56	510	(17)
	R6: AATTGATTCTTTAGCATCTGG			
***cpd***	F7: TGGTGGGTTATTTTTCAATTC	56	782	(17)
	R7: TACGGCTCTGGCTTACTA			
***ace***	F8: GGAATGACCGAGAACGATGGC	56	616	(17)
	R8: GCTTGATGTTGGCCTGCTTCCG			
***cyl*A**	F9: ACTCGGGGATTGATAGGC	56	688	(19)
	R9: GCTGCTAAAGCTGCGCTT			
***hyl***	F10: ACAGAAGAGCTGCAGGAAATG	56	276	(19)
	R10: GACTGACGTCCAAGTTTCCAA			

**Antibiotic susceptibility testing**: Applying Mueller-Hinton agar (Merck, Co., Germany) and following the guidelines of Clinical and Laboratory Standards Institute (CLSI 2020), we used the Kirby-Bauer disk diffusion technique to assess the antibiotic resistance patterns of isolated *E. faecalis* (n= 40) and *E. faecium* (n= 8) (18). One of the following antibiotics (all from Mast Diagnostics, Mast group Ltd, Merseyside, UK) was added: gentamicin (GM, 120 µg), ampicillin (AMP, 10 µg), vancomycin (VAN, 30 µg), erythromycin (ERY, 15 µg), doxycycline (DXT, 30 µg), streptomycin (S, 300 µg), ciprofloxacin (CIP, 5 µg), linezolid (LZD, 30 µg), teicoplanin (TEC, 30 µg), penicillin G (PG, 10 units), rifampicin (RP 5 µg), and quinupristin/dalfopristin (Qui/Dal, 15 µg). As the standard control strain, we employed *E. faecalis* ATCC 29212.

**Screening for virulence genes**: Applying specific primers (Table 1), multiplex PCR reactions were performed to detect *gel*E, *esp, ace, asa*1, *cpd, hyl* and *cyl*A genes. The PCR assays performed have been previously described by other researchers (17, 19). For visual assessment, after electrophoresis on ethidium bromide-stained 1.5 % agarose gel, the amplicons were screened under UV.

**Statistical analysis**: SPSS 22 software was used for statistical analyses. The data were expressed as percentages. For comparing prevalence, the Pearson's chi-square test was used considering a significance level of P <0.05.

## Results

In the present research, 48(96 %) of 50 cheese samples analyzed were found to be positive for enterococci. All enterococcal isolates were assessed using the multiplex PCR to characterize *E. faecalis* and *E. faecium*. Among all isolates, the majority were *E. faecalis* (40 out of 48, 83.33 %). *E. faecium* constituted 8 out of 48 strains (16.67 %). In total, 42.5 % (17/40) and 57.5 % (23/40) of *E. faecalis* and 75 % (6/8) and 25 % (2/8) of *E. faecium* samples belonged to Urmia and Tabriz isolates, respectively. The prevalence of enterococci ranged from 1.1×10^5^ to 9.7×10^4^ CFU/g, and 1.1×10^3^ to 9.8×10^3^ CFU/g in Urmia and Tabriz samples, respectively.

The resistance profiles of the strains are shown in Table 2. In comparison with *E. faecium*, antibiotic resistance was more common among *E. faecalis* strains. The most common patterns were resistance to rifampicin 38 (79.2 %), which was followed by quinupristin/dalfopristin 33 (68.75 %). All the strains were found to be sensitive to teicoplanin, streptomycin, linezolid, gentamicin, and vancomycin. Also, none of the *E. faecium* bacteria showed resistance to quinupristin/dalfopristin, penicillin and ampicillin (Table 2).

**Table 2:** Antibiotic susceptibility profiles of *E. faecalis* and *E. faecium* isolates from traditional cheeses

Antibiotic/Interpretive criteria	*E. faecalis* 40 (83.33 %)			*E. faecium* 8 (16.67 %)		

	Susceptible (%)	Intermediate (%)	Resistant (%)	Susceptible (%)	Intermediate (%)	Resistant (%)
**VAN**	35 (87.5)	5 (12.5)	0 (0)	8 (100)	0 (0)	0 (0)
**GM120**	40 (100)	0 (0)	0 (0)	8 (100)	0 (0)	0 (0)
**ERY**	2 (5)	29 (72.5)	9 (22.5)	2 (25)	1 (12.5)	5 (62.5)
**S**	40 (100)	0 (0)	0 (0)	8 (100)	0 (0)	0 (0)
**LZD**	25 (62.5)	15 (37.5)	0 (0)	7 (87.5)	1 (12.5)	0 (0)
**Qui/Dal**	2 (5)	5 (12.5)	33 (82.5)	7 (87.5)	1 (12.5)	0 (0)
**TEC**	40 (100)	0 (0)	0 (0)	8 (100)	0 (0)	0 (0)
**PG**	39 (97.5)	0 (0)	1 (2.5)	8 (100)	0 (0)	0 (0)
**AMP**	39 (97.5)	0 (0)	1 (2.5)	8 (100)	0 (0)	0 (0)
**RP**	1 (2.5)	7 (17.5)	32 (80)	2 (25)	0 (0)	6 (75)
**DXT**	14 (35)	0 (0)	26 (65)	7 (87.5)	0 (0)	1 (12.5)
**CIP**	25 (62.5)	0 (0)	15 (37.5)	2 (25)	0 (0)	6 (75)

Abbreviations: VAN, vancomycin; GM120, gentamicin120; ERY, erythromycin; S, streptomycin; LZD, linezolid; Qui/Dal, quinupristin/dalfopristin; TEC, teicoplanin; PG, penicillin G; AMP, ampicillin; RP, rifampicin; DXT, doxycycline; CIP, ciprofloxacin.

**Figure 1:**
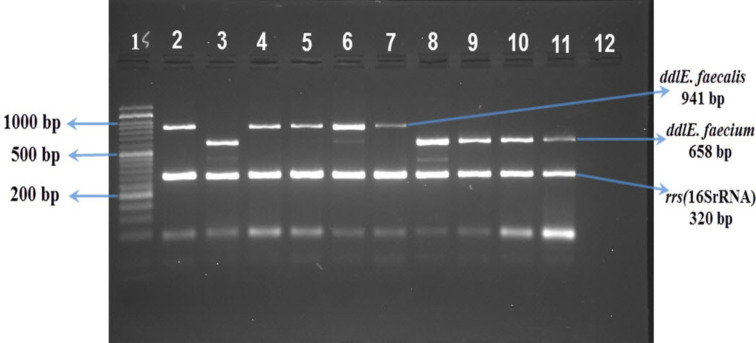
Lane 1, 50bp size marker; Lane 2, positive control (E. faecalis, ddl and rrs [16SrRNA] genes); Lane 3, positive control (E. faecium, ddl and rrs [16SrRNA] genes); Lanes 4–7, E. faecalis isolated from traditional cheese; Lanes 8–11, E. faecium isolated from traditional cheese; Lane 12, negative control without DNA

**Figure 2:**
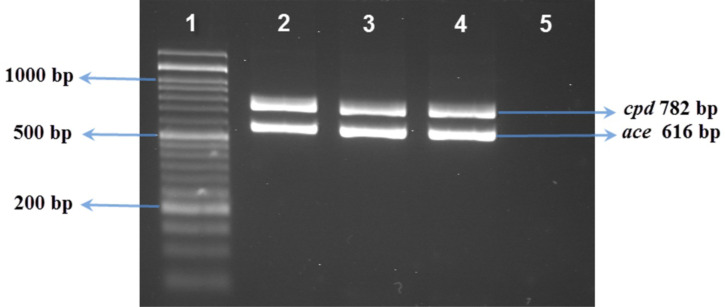
Lane 1, 50bp size marker; Lane 2, positive control for cpd and ace genes; Lanes 3, 4, Enterococcus strain isolated from traditional cheese; Lane5, negative control without DNA.

The most prevalent virulence genes expressed in *E. faecalis* isolates were *cpd* (100%), *ace* (92.5%), and *gel*E (87.5 %). In *E. faecium* isolates, *gel*E (100 %) and *asa*1 (75 %) were the most commonly detected genes (Table 3). Among *E. faecalis* isolates, *asa*1^+^, *gel*E^+^, *cpd*^+^, *ace*^+^ genotype was the most common multiple virulence genes pattern (Table 4). Overall, the multiple virulence genes genotype was more common in *E. faecalis* compared with *E. faecium* isolates (Table 4). PCR assay and gel electrophoresis used for the detection of genus and species and virulence genes are shown in Figures 1 and 2.

**Table 3:** Virulence gene positive isolates (N= 48)

Species	*gel*E (%)	*esp* (%)	*ace* (%)	*hyl* (%)	*cyl*A (%)	*cpd* (%)	*asa*1 (%)
***E. faecalis*** **(n= 40)**	35 (87.5)	1 (2.5)	37 (92.5)	0 (0)	6 (15)	40 (100)	29 (72.5)
***E. faecium*** **(n= 8)**	8 (100)	4 (50)	5 (62.5)	4 (50)	0 (0)	5 (62.5)	6 (75)
**Total= 48**	43 (89.59)	5 (10.42)	42 (87.50)	4 (8.33)	6 (12.50)	45 (93.75)	35 (72.92)

**Table 4:** Multiple virulence genes patterns in *E. faecalis* and *E. faecium* isolates

Pattern	Virulence genes	*E. faecalis* 40 (83.33 %)	*E. faecium* 8 (16.67 %)	Total 48 (100 %)
**A**	*asa*1^+^, *gel*E^+^,*cpd*^+^, *ace*^+^	19 (47.5)	2 (25)	21 (43.75)
**B**	*gel*E^+^,*cpd*^+^, *ace*^+^	9 (22.5)	1 (12.5)	10 (20.83)
**C**	*asa*1^+^,*cpd*^+^, *ace*^+^	5 (12.5)	0 (0)	5 (10.42)
**D**	*asa*1^+^,*gel*E^+^, *ace*^+^,*cpd*^+^, *cylA*^+^	4 (10)	0 (0)	4 (8.33)
**E**	*gel*E^+^,*asa*1^+^, *esp*^+^,*hyl*^+^	3 (7.5)	0 (0)	3 (6.25)
**F**	*gel*E^+^,*asa*1^+^,*cpd*^+^	2 (5)	0 (0)	2 (4.17)
**G**	*gel*E^+^,*esp*^+^, *ace*^+^,*cpd*^+^	1 (2.5)	1 (12.5)	1 (2.01)

## Discussion

For the first time, the frequency of *Enterococcus* spp. in the traditional cheese produced in two major cities in northwestern Iran was investigated in this study. Furthermore, virulence genes and antibiotic resistance patterns of *E. faecium* and *E. faecalis* obtained from these samples were investigated.

Overall, 48 of the analyzed cheese samples (96 %, 48/50) were contaminated with enterococci. According to the enumeration results, the prevalence rate of *Enterococcus* spp. among Urmia samples was higher than that of Tabriz samples. The findings of the present study demonstrated that enterococci bacteria were common in traditional cheese samples, which was in agreement with the findings of another study (1). These observations show that enterococci should be considered among the dominant bacterial populations of cheese. The prevalence of enterococci in samples ranged from 1.1×10^5^ to 9.7×10^4^ CFU/g, and 1.1×10^3^ to 9.8×10^3^ CFU/g in Urmia and Tabriz samples, respectively; Actually, the quantities and types of bacteria existing in the traditional cheeses have a wide range (2–4). The aforementioned prevalence seems to be specific to the types of traditional cheeses by special processing steps (4).

Based on the multiplex PCR analysis, the prevalence of *E. faecalis* (83.33 %) was significantly higher compared with *E. faecium* (16.67 %). Furthermore, while *E. faecalis* was the dominant species among Tabriz samples, *E. faecium* was more common species among Urmia samples. Other studies have also confirmed the presence of these bacteria in cheese (1, 20). In parallel, considerably higher detection rates of *Enterococcus* spp. have been reported by a number of other studies, reporting that *E. faecalis* was the most prevalent enterococcal bacterium in different cheese samples (1, 21, 22). Regarding the source of enterococci, it has been suggested that both cheese production equipment and cheese makers themselves can be regarded as the main pools of these bacteria (22–24). Also, the dominant presence of these bacteria may be partly explained by their compatibility with extreme salinity and resistance to heat during cheese making (2,25).

Antibiotic resistance must be regarded as an essential determinant when assessing enterococci safety profile (26). In the present study, antibiotic resistance assessment of the isolated bacteria revealed a higher resistance rate in *E. faecalis* compared with *E. faecium* species. Our observations were in accordance with the previous studies, reporting that *E. faecalis* samples were more resistant to antimicrobial agents in comparison with *E. faecium* isolates (13, 20). In the present study, enterococci showed low level antibiotic resistance, and nearly all the isolated samples were susceptible to currently available and in used antibiotics including vancomycin, gentamicin, teicoplanin, linezolid, streptomycin, penicillin, and ampicillin. The lower incidence antibiotic resistance in our study was in line with the results of previous reports, indicating the low diffusion of antibiotic resistance in food-derived enterococci (10), which is of critical importance for the health of consumers (27). Overall, low antibiotic resistance among enterococci is an advantageous feature in favor of their usage in food production industries.

The highest antibiotic resistance rate among enterococci was recorded against rifampicin (79.2 %). The alarming situation was the relatively high prevalence of quinupristin/dalfopristin (68.75 %) among isolates; interestingly, this antibiotic has recently been added to the list of health care system. The unexpected quinupristin/dalfopristin resistance among clinical specimens has been reported earlier in the area, although the drug has not been used yet in hospital settings (28).

Enterococci can be potential pathogens, which is a global health concern, and in fact, these bacteria have been reported to be associated with some infections (7). However, only a number of *Enterococcus* spp., particularly *E. faecalis* and *E. faecium*, have been recognized with clinically important virulence factors (29, 30).

In the present research, there was a significant difference in the detection rate of various virulence genes comparing *E. faecium* and *E. faecalis* strains (*P* < 0.05). In this regard, *cyl*A, *ace, asa*1, and *gel*E virulence genes were more commonly detected in *E. faecalis* than *E. faecium* isolates, corroborating the results of previous reports (9, 10, 13, 31).

In our study, the most prevalent (100 %) virulence gene among *E. faecalis* samples was *cpd*. In agreement with this observation, a considerably high prevalence of sex pheromone determinants (*ccf, cob*, and *cpd*) was noted in *E. faecalis* strains in another report (20). Furthermore, the *esp* gene was identified in half of *E. faecium* samples and 2.5% of *E. faecalis* isolates (*P* < 0.05). Nevertheless, the *esp* gene was detected in a relatively high number of *E. faecalis* strains in several other studies (9,17,20,32). We also identified the *hyl* gene in half of *E. faecium* isolates, which was inconsistent with the findings of another research reporting that the *hyl* gene was absent in all analyzed *E. faecium* and *E. faecalis* species (13). It is noteworthy that various sources such as industries, poultries, foods, animals, and plants can contribute in the development of bacterial antibiotic resistance in hospitals and humans (33,34).

To conclude, our findings demonstrated a high prevalence of *Enterococcus* spp. in the traditional cheese samples collected from two major cities (Tabriz and Urmia) in northwestern Iran. This is important as these bacterial strains can harbor antibiotic resistance and potential virulence traits. Meanwhile, the most prevalent isolated species was *E. faecalis*. Although enterococci species are generally known as nonpathogenic bacteria, emerging antibiotic resistant strains and expressing various virulence factors can confer them highly pathogenic and infectious features. Therefore, the presence of enterococci in cheese and other dairy products must be regarded as a potential public health threat. In the current study, we presented the essential molecular and phenotypic parameters required to investigate enterococci in traditional cheese. These criteria are suitable to be used by food quality and sanitary control laboratories to ensure the safety of nutritional products.

A limitation of this study is that the numbers samples were relatively small, but the results were not inconsistent with other studies. The limitation was related to small number of main manufacturers of the traditional cheeses who distributed the cheeses in the Urmia and Tabriz.
